# Dizziness in community-dewelling older adults: a population-based study

**DOI:** 10.1590/S1808-86942011000600003

**Published:** 2015-10-19

**Authors:** Suzana Albuquerque de Moraes, Wuber Jefferson de Souza Soares, Rosilene Andrade Silva Rodrigues, Waléria Christiane Rezende Fett, Eduardo Ferriolli, Monica Rodrigues Perracini

**Affiliations:** 1 Physical therapist, specialist, master's degree student in physical therapy, São Paulo City University (Universidade Cidade de São Paulo, UNICID); 2 Physical therapist, master's degree in physical therapy, São Paulo City University (UNICID). Higher education level technician of the Health Secretariat of Mato Grosso state; 3 Physical therapist, master's degree student, Mato Grosso Federal University (UFMT); 4 Physical educator, doctoral degree in medical science, Ribeirão Preto Medical School, Sao Paulo University (Faculdade de Medicina de Ribeirão Preto, USP). Faculty member of the Physical Education School, UFMT; 5 Physician, doctoral degree in medicine, São Paulo University (USP). Faculty member of the Internal Medicine Department, Ribeirão Preto Medical School, USP; 6 Doctoral degree in science, Sao Paulo Federal University (Universidade Federal de São Paulo, UNIFESP). Faculty member of the Master's Degree Program in Physical Therapy, UNICID

**Keywords:** aged, dizziness, health of the elderly, vertigo

## Abstract

**Abstract:**

Dizziness is a common complaint among older adults.

**Aim:**

To identify the prevalence of dizziness and its related factors in a sample of community-dwelling older adults.

**Methods:**

A prospective population-based study with a representative sample of older adults aged 65 years and over. A multidimensional questionnaire and a battery of measures were used for assessing physical function.

**Results:**

The prevalence of dizziness was 45%. Vertigo was found in 70.4% of older adults with dizziness and 43.8% of them referred vertigo crises along life. A significant association was found between dizziness and female gender (*p*=0.004), memory difficulties complaints (*p*=0.015), bad health perception (*p*=0.001), depression (*p*<0.0001), five or more comorbidities (*p*=0.021), self-reported fatigue (*p*<0.0001), recurrent falls (*p*=0.001), excessive sleepiness (*p*=0.003), fear of falling (*p*<0.0001), left leg unipedal stance (*p*=0.002) and Short Performance Physical Battery score (*p*=0.009).

**Conclusion:**

Dizziness is a common complaint among older adults and it is associated with limiting clinical conditions, such as depression, fatigue, excessive sleepiness and impaired memory. It is highlighted the association between dizziness and recurrent falls, fear of falling and lower performance in physical function tests, which can restrict independence. These data suggest the importance of a multifactorial approach to dizziness in older adults.

## INTRODUCTION

Dizziness is a frequent complaint in the elderly population, and its prevalence increases significantly with age^1^, ^2^, ^3^, ^4^, ^5^. Recent population studies have reported a prevalence ranging from 11.0% to 32.5%^6^, ^7^, ^8^. A longitudinal study of elderly patients aged 65 years and over found that the prevalence of dizziness within the past six months among subjects aged 70 years was 27%, and that among patients aged 90 years or more it was 54%^4^. The incidence was 83.3 per 1,000 inhabitants among elderly patients monitored in primary care; it was 67.8 per 1,000 inhabitants among elderly patients aged from 65 to 74 years, and increased to 108.4 per 1,000 inhabitants among patients aged 85 years or more^9^. The prevalence of dizziness is higher is women than men (the male/female incidence in one year is 1:2.7)^2^. Dizziness and vertigo are associated with an increased risk of falls and loss of function, which may results in a substantial loss of independence and worse quality of life in this population group^1,^^10^, ^11^, ^12^.

The rate of falls within the past year in elderly patients with vestibular disease is significantly higher – about 50% – compared to the reported rate in community-dwelling elderly patients (about 30%)^13^. Falls within the past three months were reported by 31% of elderly subjects with dizziness compared to 15% in elderly subjects not complaining of dizziness^5^. The risk of falls is increased in adults and elderly persons with vestibular dysfunction^1^. The chance of falling was about three times higher among subjects with dizziness compared to those without this complaint in a two-year follow-up of community-dwelling elderly subjects (OR – 3.24; CI – 95%, 2.11-4.99)^4^. Dizziness and vertigo are risk factors for falls and severe injury such as fractures^14^,^15^. Vestibular dysfunction limits postural control, leading to instability and misaligned posture; this explains why dizziness may be a predisposing factor for falls^16^.

Elderly individuals with vestibular dysfunction may find it difficult to perform activities of daily life that require rapid head movements^17^. Dizziness can make it difficult for patients with vestibular dysfunction to carry out activities of daily life even while symptoms regress^18^. Among elderly patients with chronic vestibular dysfunction, 49% reported difficulties in carrying out seven of more activities, 26% in one to three activities, and 19% in four to six activities; only 7% said that they could carry out activities of daily life without any difficulties. The activities that discriminated elderly individuals with more limitations were walking close to home, taking a shower, and shopping, which require coordinated head and eye movements and demand more of the vestibular system^12^.

There is an association between chronic dizziness and symptoms of depression, self-reported poor health, and restriction in social activities^19^,^20^. About 56% of elderly patients with vestibular diseases and 42% of elderly individuals with complaints of dizziness score positively for symptoms of depression^5^,^21^. Associated factors are female gender, memory and concentration disorders, insomnia, hypoacusis, and poor vision^21^. Dizziness in elderly subjects is also associated with lack of physical activity and poor emotional and physical quality of life. There is a strong association between depression and poor quality of life in elderly individuals with dizziness; there is evidence in the literature that neuro-otologic dysfunction is related with anxiety and psychological problems, which in turn may increase the intensity of dizziness^5^.

Dizziness has been characterized as a geriatric symptom, a multifactor condition resulting from the cumulative effect of losses in several systems, which increase the vulnerability of elderly individuals^10^. A few medical conditions have been associated with dizziness, such as anxiety, depression, impaired hearing, using five or more medications, postural hypotension, disordered body balance, and a history of acute myocardial infarction^10^. A two-year longitudinal follow-up study revealed that the main predictors of dizziness as a complaint in elderly individuals were: age, female gender, cardiovascular disease, osteoporosis, depression, disordered sleep, memory disorders, poor vision, urinary incontinence, three or more co-morbidities, use of four or more medications, poor perception of health, falls, and mobility issues^4^. Several sensations associated with dizziness as a complaint have been reported in the literature, such as loss of balance (64.5%), near syncope (11.4%), turning around (41%), and its manifestation as single or multiple conditions daily or weekly in 68% of cases^4^,^20^.

Because the elderly population has increased, it has been estimated that the number of elderly individuals with dizziness and vertigo has also increased. Identifying the profile of elderly patients with dizziness as a complaint will help plan specific interventions for this age group. The purpose of this study was to investigate the prevalence of dizziness as a complaint and its associated factors in a population of community-dwelling elderly individuals, and to characterize dizziness in terms of its course, duration, sensation of falling, unbalance, periodicity, intensity, associated symptoms, and triggering functional activities and movements.

## MATERIAL AND METHODS

An exploratory cross-sectional epidemiological study was carried out as a sub-project of the Study Network on the Frailty of Brazilian Elderly Individuals (*Rede de Estudos de Fragilidade de Idosos Brasileiros* or *Rede FIBRA* – FIBRA network*)*, which is a multicenter multidisciplinary population based network that aims to study the features and prevalence of the biological frailty syndrome according to Fried et al.'s^22^ proposed phenotype. It encompasses 17 Brazilian sites selected by quota sampling based on human development indices.

The sample comprised 391 elderly subjects of both sexes aged 65 years or more that resided in the city of Cuiaba, MT, who were assessed from March 2009 to March 2010.

The exclusion criteria consisted of Ferrucci et al's^23^ recommendations: 1) presence of memory, attention, spatial and time orientation, and communication deficits suggesting severe cognitive disorders as assessed by the Mini-Mental State test^24^, ^25^, ^26^ according to the following scores^25^ – 17 for illiterates, 22 for elderly subjects with 1 to 4 years schooling, 23 for elderly subjects with 5 to 8 years schooling, and 26 for subjects with more than 9 years schooling, less a standard deviation; 2) permanent or temporary inability to walk – walking sticks or walkers are allowed, but not wheelchairs; 3) localized loss of strength and aphasia because of a stroke; 4) severe loss of movement, speech, or affect associated with severe or unstable Parkinson's disease; 5) severe hearing loss and visual deficits that make it difficult to communicate; 6) terminal stage patients.

Fifteen census regions were visited in Cuiaba - 513 elderly individuals were enrolled. Twenty-eight subjects were excluded based on the exclusion criteria. Ninety-four individuals quit after the questionnaires were applied.

**Figure 1:**
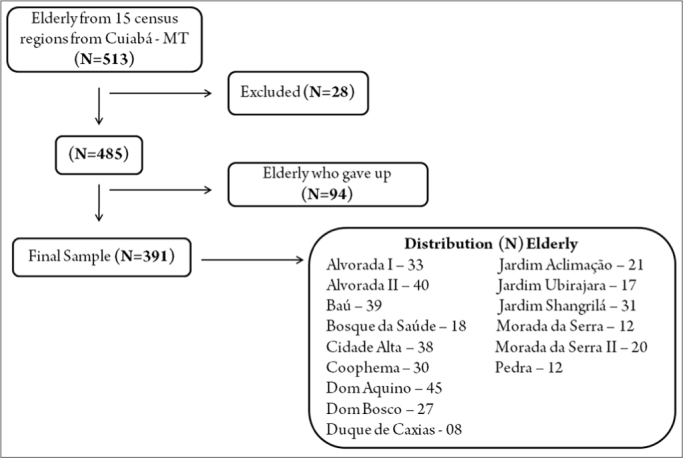
Study sample flowchart.

Figure 1 (below) shows the study sample flowchart.

Subjects were informed about the purpose and procedures of the study and signed a free informed consent form. The approval protocol number of the institutional review board of the Ribeirão Preto Clinic Hospital was 5018/2007.

Elderly subjects were evaluated in two steps, as follows: the first consisted of a semi-structured interview – common to all participating centers of the FIBRA network – carried out by trained interviewers in a 40 to 120 minute session at the subject's household; the second step consisted of a data-gathering session by trained personnel on the physical and functional status, lasting 20 to 30 minutes, comprising a battery of ten standard function tests and anthropometric data, carried out in public schools, community centers, and city health centers close to the households of subjects.

The following variables were included: age, gender, whether living alone, education level, self-reported diseases (heart conditions, arterial hypertension, stroke, diabetes, depression), obesity^27^, number of diseases and medications, hospital stays within the past year, subjective perception of vision and hearing, smoking, memory and sleep disorders, functional performance as assessed by Lawton's instrumental activities of daily living (IADL)^28^ and advanced activities of daily living (AADL)^29^, self-efficacy for falls (Falls Efficacy Scale International or FESI)^30^,^31^, symptoms of depression as assessed by the Geriatric Depression Scale (GDS-15)^32^,^33^, self-reported fatigue (Center for Epidemiological Studies Depression Scale or CES-D)^34^, a subjective perception of health, and the extended inquiry about falls, sleepiness (Epworth sleepiness scale)^35^, and dizziness.

Dizziness was investigated based on a self-reported presence of this symptom within the past year; the question was: “Did you feel dizziness in the past year?” Elderly subjects were asked to think of dizziness as any sensation of rotating, gyrating, feeling emptiness or weight in the head, dizziness itself, and fluctuation. The question for vertigo was: “When you feel dizziness, do you feel your body or head going round, or what is around you going round?”

The question for characterizing dizziness was: progression time, duration, presence of falling sensation, unbalance or instability while walking, periodicity, presence of dizziness in 14 situations and/or activities: standing from decubitus, turning the head, turning the body from a sitting or standing position, standing from a sitting position, walking, while anxious, head in specific positions, sitting still, changing position on the bed, while exercising, laying on a side, when nervous, after eating, not having breakfast, and associated symptoms (tinnitus, headache, memory and concentration deficits, hypersensitivity to sounds, nausea, vomiting, sweating, anxiety, feeling imminent syncope, pressure, fear, insomnia, oscillopsy, or hearing loss).

The physical-functional tests were: timed up and go test^36^,^37^, five step test^38^, right and left one-leg balance time^39^, short physical performance battery (SPPB)^40^, ^41^, ^42^, ^43^, and habitual gait velocity.

A descriptive analysis comprised simple frequency and central tendency measures, with a 95% confidence interval. The Kolmorogov-Smirnov test was applied to check compliance with a normal distribution. The chi-square test or Fisher's exact test for categorical variables and the “t” test or the Mann-Whitney test for quantitative variables were applied to compare the groups with or without a complaint of dizziness; the significance level was α<0.05. The SPSS^®^ version 13.0 for Windows software was used for the statistics.

## RESULTS

The prevalence of dizziness was 45.0% (N=176). Of these, 31.7% reported dizziness within the past two months. Rotating dizziness was reported by 70.4% of elderly subjects; 43.8% of subjects reported past vertigo crises at some point in their lives.

The mean age of elderly subjects with dizziness was 72.26 ± 5.97 years; it was 72.55 ± 6.16 years in subjects not reporting dizziness. This difference was not statistically significant (*p*=0.686). About 70% of subjects complaining of dizziness were aged from 65 to 74 years.

Women were 71.6% of the sample with dizziness; men were 28.4% (*p*=0.002). Elderly subjects with a complaint of dizziness had a higher rate of more than five co-morbidities compared to those not complaining of dizziness (60.9% vs 39.1%); they also scored positively in depression screening (62.7% vs 37.3%), excessive sleepiness (60.5% vs 39.5%), fatigue (60.8% vs 39.2%), poorer perception of health (53.3% vs 46.7%), memory disorders (51.6% vs 48.4%), and recurrent falls within the past year (64.1% vs 35.9%) (Table 1).

**Table 1:** Characterization of the sample of elderly subjects with and without a complaint of dizziness (N=391) in the city of Cuiaba, Mato Grosso.

Variables	Dizziness Yes (N=176) %(N)	Dizziness No (N=215) %(N)	*p*-value
Female	50.6 (126)	49.4 (123)	0.004
Mel	35.2 (50)	64.8 (92)	0.599
Up to 4 years education	47.1 (123)	52.9 (138)	0.079
Living alone	54.1 (33)	45.9 (28)	0.063
Heart disease	55.7 (39)	44.3 (31)	0.323
Arterial hypertension	46.7 (127)	53.3 (145)	0.862
Stroke	47.1 (8)	52.9 (9)	0.901
Diabetes	43.9 (36)	56.1 (46)	0.015
Memory difficulties	51.6 (96)	48.4 (90)	0.147
Difficult to sleep	49.7 (78)	50.3 (79)	0.539
Poor hearing	48.2 (41)	51.8 (44)	0.127
Poor vision	48.8 (100)	51.2 (105)	0.898
Hospital stay	44.0 (33)	56.0 (42)	0.266
Smoking	53.3 (24)	46.7 (21)	0.001
Poor perception of health	53.3 (16)	46.7 (14)	0.350
Unsatisfactory life	41.1(74)	51.9(80)	<0.001
Positive GDS score	62.7 (74)	37.3 (44)	0.021
5 or more diseases	60.9 (14)	39.1 (9)	<0.001
Self-reported fatigue	60.8 (73)	39.2 (47)	0.058
Falls	51.4 (75)	48.6 (71)	0.001
Recurring falls	64.1 (41)	35.9 (23)	0.003
Excessive sleepiness	60.5 (46)	39.5 (30)	0.127
AADL (≥ 10 of 12)*	52.0 (51)	48.0 (47)	0.476
IADL (≥ 3 of 7)*	41.5 (39)	58.5 (55)	0.859
Obesity (≥ 27 kg/ cm2)	43.3 (88)	53.7 (102)	0.982
Polypharmacy (≥ 4 med)	47.1 (41)	52.9 (46)	0,982

GDS (Geriatric Depression Scale).

AADL (Advanced Activities of Daily Living).

IADL (Instrumental Activities of Daily Living).

* Cutoff point – percentile 75.

Most elderly subjects (68.7%) reported dizziness progressing for more than one year; of these 24.4% reported a complaint of dizziness for more than 5 years. Dizziness was constant in 2.8% of subjects; it lasted several days in 4.5% of subjects. It lasted less than one minute in 54.0% of subjects, and from one minute to two hours in 30.7% of the sample.

About 70% of subjects with dizziness reported a falling sensation, and half reported unbalance or instability while walking.

Dizziness was sporadic in 71.6% of subjects, monthly in 11.4% of subjects, and daily in 13.1% of subjects.

The dizziness intensity score on a visual analog scale was less than 5 points in 51.8% of the sample; the score was above 5 points in 18.7% of the sample. The intensity score was equal to or higher than 7 in 13.6% of subjects.

Half of the elderly subjects with dizziness said that standing from a lying position was the initiating cause of dizziness; 48.3% reported turning the head as the triggering factor, and 38.1% mentioned standing from a sitting position. The most frequent associated symptoms was tinnitus (18.7%), followed by sweating, pallor and tachycardia (16.6%), feeling anxiety (16.4%), a sensation of blocked ear (15.6%), and headache (14.8%). Table 2 shows the feature of dizziness relative to the positions, triggering activities and associated symptoms.

**Table 2:** Characterization of dizziness relative to activities and main triggering positions and symptoms in community-dwelling elderly individuals.

Variables	Dizziness Yes (N=176) % (N)
Standing from a lying position	50.0 (88)
Turning the head	48.3 (85)
Turning the body from a sitting or standing position	30.7 (54)
Standing from a sitting position	38.1 (67)
Walking	17.9 (70)
When anxious	12.3 (48)
Head in specific position	6.4 (25)
Sitting still	3.6 (14)
Changing position in the bed	11.8 (46)
During exercise	11.0 (43)
Lying sideways	5.4 (21)
When nervous / stressed	12.8 (50)
After eating	2.6 (10)
Not having breakfast	7.7 (30)
Tinnitus/Hissing/Sensation of waterfall noise	18.7 (73)
Headache	14.8 (58)
Memory and concentration disorders	14.1 (55)
Excessive sensitivity to sounds	13.0 (51)
Nausea	10.2 (40)
Vomiting	7.2 (28)
Sweating/Pallor/Tachycardia	16.6 (65)
Anxiety	16.4 (64)
Sensation of imminent fainting	12.0 (47)
Sensation of blocked ear	15.6 (61)
Feeling fear	14.1 (55)
Insomnia	13.8 (54)
Oscillopsy	9.2 (36)
Hearing loss	9.0 (35)

Table 3 shows gait and movement-related physical and functional performance tests, and fear of falling in elderly subjects with and without a complaint of dizziness. Subjects with dizziness, compared to those that do not complain of dizziness, have a poorer sensation of self-efficacy to avoid falls (*p*<0.001), are more concerned about the possibility of falls, lower left one-leg balance time (*p*=0.002), and poorer lower limb functional performance (*p*=0.009) as assessed in the SPPB.

**Table 3:** Characterization of the physical and functional performance and fear of falling in community-dwelling elderly subjects with and without a complaint of dizziness (N=391).

Variables	Dizziness Yes (N=176)			Dizziness No (N=215)			*p*-value
	Mean (DP)	Median	95% CI	Mean (SD)	Median	95%CI	
Fear of falling (FESI. pts)	30.58 (11.20)	29.0	28.91 - 32.25	27.93 (12.74)	23.0	26.21 - 29.64	<0.0001^a^
TUG (s)	12.28 (5.72)	11.14	11.43 - 13.13	12.18 (7.85)	10.85	12.18 - 11.13	0.890^b^
Five Step Test (s)	16.55 (7.28)	15.26	15.46 - 17.63	16.03 (6.71)	15.67	15.13 - 16.94	0.470^b^
Right one-leg balance (s)	12.70 (15.94)	6.26	10.33 - 15.07	15.74 (17.79)	9.19	13.34 - 18.13	0.064^a^
Left one-leg balance (s)	11.94 (16r04)	4.96	9.55 - 14.34	15.34 (17.32)	8.75	13.01 - 17.67	0.002^a^
SPPB (pts)	8.64 (2.31)	9.0	8.30 - 8.99	9.66 (6.46)	10.0	8.79 – 10.53	0.009^a^
HGS (m/s)	0.93 (0.28)	0.92	0.89 - 0.98	0.98 (0.32)	0.96	0.93 - 1.02	0.163^b^

FESI (Falls Efficacy Scale International); TUG (Timed Up and Go Test); SPPB (Short Physical Performance Battery); HGS (Habitual Gait Speed) pts (points); s (seconds); m/s (meters per second).

a Mann-Whitney test

a T-test.

## DISCUSSION

In this study, the prevalence of dizziness as a complaint within the last year was 45%; 70.4% of these reported rotating dizziness. Most published studies have reported a prevalence around 30%^6^,^10^,^44^. Gasmman & Rupprecht^4^ reported 29.2% in a longitudinal cohort study of 620 elderly individuals aged 65 years or more. Tamber et al.^45^ reported a prevalence of 36.2% at age 75 years in a cross-sectional cohort study of 3,352 elderly subjects (the Oslo Health Study), which was similar to Gopinath et al.'s^46^ findings in a study of 2,751 subjects aged 50 years and above in the Blue Mountains Hearing Study. Cabral et al.^47^ evaluated dizziness as a complaint in elderly individuals that were monitored at a primary care center and found that the prevalence was 47%. Only one study reported a low prevalence (11.1%); an explanation was the question posed to participants: “With what frequency do you have dizziness when walking over a flat surface?” – this restricts other sensations and activities related to dizziness. Dizziness is an important symptom among the elderly. The term, however, is considered non-specific because it describes a wide range of sensations, such as feeling the head empty, fluctuation, stunning, dizziness, and others. There is no consensus on the definition of dizziness for population and epidemiological studies^4^,^45^. It is considered a wide range of sensations related to bodily orientation in space. Vertigo is considered a subtype of dizziness; it is defined as an illusion of movement, commonly a rotating sensation^10^,^46^. Dizziness as a complaint is approached from several angles in questionnaires – face to face interviews, mail, or telephone^4^,^6^,^10,^^44^, ^45^, ^46^. Questions routinely used in published studies are: “Do you have any problem such as vertigo, dizziness, poor balance, or instability in general?^44^ “Have you experienced any sensation of dizziness or instability within the past year?”^46^ “Have you had dizziness within the past six months?”^4^ “Below is a list of issues. Have you experienced any of them within the past week?” (one of the 10 issues was dizziness)^46^; “Within the past two months have you felt giddy, unstable, or as if you were rotating or moving, with your head empty, or fainting?”^10^ Interviewers in the present study were trained to ask about dizziness in more detail by describing to the interviewees several sensations which could be characterized as dizziness; the goal was to avoid sub-reports due to lack of knowledge or information. We also believe that face to face interviews may have helped the elderly subjects to clarify their sensations and to define whether they were in the category of dizziness.

The most common functional activities or movements associated with dizziness were (Hurvitz, 2000 #93): standing from a lying position, turning the head, and standing from a sitting position. The first two were also the most frequent in Tinetti et al.'s^10^ study. Standing from a lying position and walking were more frequent in Gassmann & Rupprecht's^4^ study. A study that assessed elderly patients with vestibular diseases found that turning the head and keeping the head in a specific position were the most common movements or positions associated with dizziness^20^.

The complaint of dizziness was more common in females compared to males (50.6% vs. 35.2%), which is supported by several studies^4^,^9^,^10^,^13^,^20^,^21^,^46^,^48^. Some studies suggest a correlation between age and dizziness, with a higher prevalence in older age groups^4^,^9^. Other, as in the present study, found no independent association between age and a complaint of dizziness^8^,^10^. Stevens et al.^8^ found no significant age difference in elderly patients with and without dizziness in the English Longitudinal Study of Aging (ELSA); there was an independent relationship between age and disordered body balance.

As expected, there was a significant association between number of diseases and dizziness. Although a nonspecific symptom in some cases, dizziness is largely related to vestibular system dysfunction, which is often multifactorial in the elderly.^4^ Subjects reporting dizziness or vertigo in a study involving of 2,751 subjects to clarify the etiology of dizziness revealed that 27.7% were of vestibular origin, 39.3% of non-vestibular origin, and 33.0% were inconclusive^46^. A Dutch study found that 39% of elderly subjects aged 65 or older who sought their family physicians because of dizziness were diagnosed as having dizziness of unknown causes – after examinations, tests and consultation with specialists. In several other studies, the prevalence of dizziness was higher in elderly patients that presented more co-morbidities^9^,^10^,^45^.

The most relevant clinical findings were depression, excessive sleepiness, exhaustion fatigue, memory disorders, and recurring falls. Depression has been associated with dizziness in several papers^4^,^8^,^10^. Tinetti et al.^10^ found that elderly individuals with more symptoms of depression in the CES-D tool had 1.36 times more chances of presenting dizziness (95% CI; 1.02-1.80) compared to those with fewer symptoms. Similarly, Gassmann & Rupprecht^4^ indicated a 3.89 higher chance (95% CI; 2.07-7.30), and Stevens et al.^8^ found a 2.17 times higher chance (95% CI; 1.56-3.01). The relationship between depression, symptoms of depression, and the effects of antidepressant use is complex. The complaint of fatigue was measured in this study by asking the following questions taken from the CES-D tool (“Did you feel you had to make an effort to deal with habitual tasks?”and “Have you left aside many of your interests and activities?”). Also, reports of memory disorders and excessive sleepiness may correlate both with dizziness and depression. Although these factors were independently associated with dizziness in the present study, our statistical analysis did not provide any estimate of the weight of these associations. A study of 120 elderly individuals with chronic vestibular syndrome suggested – in a linear regression model – that having more symptoms of depressions was associated with memory and concentration disorders, among other factors^21^.

Poor perception of health was independently associated with dizziness, which concurs with other population-based studies^4^,^46^. Elderly subjects that considered their health as poor and very poor had 4.19 times the chance (CI 95%; 2.77-6.33) of complaining of dizziness compared to those without these complaints.

In the present study, 51.4% of elderly subjects that had falls reported dizziness, and 64.1% of elderly individuals with recurring galls reported dizziness. Stevens et al.^8^ argued that dizziness was strongly associated with falls, even after adjusting for gender (odds ratio – 2.11 95%; CI 1.69-2.64). Gassmann & Rupprecht^4^ found that elderly subjects reporting falls within the past six months had a 3.24 chance of manifesting dizziness compared to elderly individuals that did not fall. Stability and postural orientation mechanisms should work adequately to avoid falls. Most disorders involving dizziness interfere with that control, and may increase the susceptibility of elderly individuals to falls. Furthermore, elderly subjects with dizziness in the present study had a poorer perception of self-efficacy in avoiding falls; they were more fearful of falls compared to elderly individuals without dizziness. A study of 200 elderly individuals aged 60 year or more that were monitored at a geriatrics outpatient clinic showed that patients with chronic dizziness had a 4.9 higher chance of manifesting fear of falling (CI 95%; 2.2-11.1) compared to elderly individuals without dizziness^48^.

Among the physical and functional tests, there was an association between dizziness and left one-leg balance time and the SPPB score, showing that elderly patients with dizziness scored significantly worse than subjects without dizziness. The leg balance test places a high demand on musculoskeletal system control and mid-lateral body stability, and has been used to identify older adults at a higher risk of falling^49^. The SPPB consists of a test battery for static equilibrium with a progressive decrease of support, usual gait speed, sitting and standing sub-test. Other studies have reported a significant association between dizziness and poor performance on static equilibrium tests similar to those used in this study^8^,^10^.

This population-based and epidemiological study was carried out using a representative sample of community-dwelling elderly subjects, and was based on a multidimensional assessment tool. The findings, therefore, are highly relevant for establishing the profile of elderly individuals presenting dizziness within the past year. Due to its cross-sectional design, however, no causal relationships could be established.

It is important to highlight the importance of screening for dizziness in the elderly community, due to its high prevalence and impact on health and function in the elderly. Dizziness is sometimes overlooked because of its complex and multifactorial etiology. However, a correct diagnosis and treatment may avoid loss of health in this population.

## CONCLUSION

Dizziness is a common complaint in elderly individuals. It is most frequently characterized as being sporadically present for at least one year and lasting a few seconds. The main reported triggering activities for dizziness are standing from a lying or sitting position and turning the head. Significantly associated factors were female gender, depression, memory disorders, poor perception of health, co-morbidities, fatigue, excessive sleepiness, fear of falling, and poor performance in physical/functional tests of body balance. These data suggest that a multifactorial approach of dizziness in elderly individuals and interventions for improving the functional capacity and preventing falls are important.
